# Infant and young child feeding practices differ by ethnicity of Vietnamese mothers

**DOI:** 10.1186/s12884-016-0995-8

**Published:** 2016-08-08

**Authors:** Tuan T. Nguyen, Phuong H. Nguyen, Nemat Hajeebhoy, Huan V. Nguyen, Edward A. Frongillo

**Affiliations:** 1 Technical Specialist in Measurement, Learning & Evaluation, Vietnam, Alive & Thrive, Alive & Thrive Project, FHI 360, 7th floor, Ha Noi Tourist Building, 18 Ly Thuong Kiet street, Ha Noi, Vietnam; 2 International Food Policy Research Institute (IFPRI), Washington, DC, USA; 3 Present Address: Bill & Melinda Gates Foundation, Seattle, WA USA; 4 Save the Children, Hanoi, Vietnam; 5 Arnold School of Public Health, University of South Carolina, Columbia, SC USA

**Keywords:** Breastfeeding, Infant and young child, Complementary feeding, Ethnicity, Vietnam

## Abstract

**Background:**

Limited studies have examined ethnic variation in breastfeeding and complementary feeding practices in developing countries. This study investigated ethnic variation in feeding practices in mothers with children 0–23 months old in Vietnam.

**Methods:**

We used data on 1875 women who came from the ethnic majority, Kinh (*n* = 989, randomly sampled from 9875 surveyed Kinh mothers, 10 % from each province) and three ethnic minorities: E De-Mnong (*n* = 309), Thai-Muong (*n* = 229) and Tay-Nung (*n* = 348). Ethnic minorities were compared with the Kinh group using logistic regression model.

**Results:**

Prevalence of breastfeeding initiation within an hour of birth was 69 % in Thai-Muong, but ~50 % in other ethnicities. In logistic regression, the prevalence of breastfeeding within one hour was lower in Tay-Nung (OR: 0.54; 95 % CI: 0.38, 0.77) than the majority Kinh. Prevalence of exclusive breastfeeding under 6 months was 18, 10, 17, and 33 % in Kinh, Thai-Muong, Tay-Nung, and E De-Mnong, respectively; compared to the majority Kinh, the prevalence was lower in Thai-Muong (OR: 0.42; 95 % CI: 0.25, 0.71) and higher in E De-Mnong (OR: 1.99; 95 % CI: 1.04, 3.82). Overall prevalence of bottle feeding in Thai-Muong and E De-Mnong (~20 %) was lower than in Kinh (~33 %): Thai-Muong (OR: 0.50; 95 % CI: 0.37, 0.68) and E De-Mnong (OR: 0.69; 95 % CI: 0.50, 0.95). Compared with Kinh (75 %), fewer ethnic minority children received minimum acceptable diets (33 % in Thai-Muong, 46 % in E De-Mnong, and 52 % in Tay-Nung; *P* < 0.05). Prevalence of minimum acceptable diet (met both dietary frequency and diversity) was lower in Thai-Muong (OR: 0.23; 95 % CI: 0.11, 0.46), Tay-Nung (OR: 0.52; 95 % CI: 0.39, 0.69), and E De-Mnong (OR: 0.55; 95 % CI: 0.33, 0.89) than the majority Kinh.

**Conclusions:**

Breastfeeding practices were suboptimal and differed by ethnicity, which suggests need for tailored interventions at multiple levels to address ethnic-specific challenges and norms. Complementary feeding practices were less optimal among ethnic minorities compared to Kinh, which suggests need for broad intervention including improved food availability, accessibility, and security.

**Electronic supplementary material:**

The online version of this article (doi:10.1186/s12884-016-0995-8) contains supplementary material, which is available to authorized users.

## Background

Race, ethnicity, and socio-economic status are associated with nutrition and health outcomes through social, physical, behavioral, and biological mechanisms [1]. For specifically infant and young child feeding (IYCF) practices, findings from studies in high-income countries suggest ethnic variation in early initiation of breastfeeding in Ireland [2] and United Kingdom [3], ever breastfed and breastfed at 6 months in the United States [4, 5], continued breastfeeding in United Kingdom [3] and the Netherlands [6], and timely introduction of solid foods in United Kingdom [7]. Potential explanations for ethnic variation in IYCF practices were cultural attitudes and norms [8], acculturation [6, 9], and underlying determinants that link with ethnicity such as demographic and socioeconomic status [9–12].

The association between ethnicity and breastfeeding practices in low- or middle-income countries might differ from that in high-income countries because of differences in socio-economic determinants and breastfeeding patterns (eg, initiation of breastfeeding and continued breastfeeding prevalence are higher). Yet, limited information about the association between ethnicity and breastfeeding practice exists in low- or middle-income countries. Also, previous studies in high-income countries [2–7] did not use the IYCF indicators recommended by the World Health Organization (WHO) [13] to examine the ethnic variation.

Vietnam is similar to many countries throughout the world in having many ethnically distinct groups: 53 ethnic minority groups account for about 15 % of the total ~ 90 million people in Vietnam. Cultural, demographic, and socioeconomic characteristics of different ethnic groups can vary substantially [14], which can affect IYCF practices and related factors. To date, however, little data on IYCF practices of different ethnic groups have been reported. Among recent national nutrition reports [15–19], only the Vietnam Multiple Indicator Cluster Survey (MICS) [17, 20] provided descriptive information on ethnic minority and disadvantaged mothers (from 52 ethnic groups). They had a higher prevalence of early initiation of breastfeeding, exclusive breastfeeding under 6 months, and continued breastfeeding at 1 and 2 years than members of ethnic majority groups (Kinh and Hoa). Reports of surveys with IYCF indicators from other countries, such as Demographic and Health Surveys (DHS) and Multiple Indicator Cluster Survey (MICS), typically combined ethnic minorities [21, 22].

To gain better understanding of the role that ethnicity plays in IYCF, we examined ethnic variation in breastfeeding and complementary feeding practices among mothers with children 0–23 months old in Vietnam. Specifically, we compared WHO recommended key IYCF practices in four Vietnam ethnic groups: Kinh, Thai-Muong, Tay-Nung, and E De-Mnong.

## Methods

### Participants

Data for this study are from an evaluation of the Alive & Thrive (A&T) project that aimed to reduce undernutrition by improving IYCF practices at large scale [23]. The study design and sample selection have been described in detail elsewhere [24–26]. Briefly, mothers were recruited using a three-stage cluster sampling technique that selected: 1) intervention and comparison districts, 2) primary sampling units (PSU, equivalent to an average-sized village) based on population-proportionate-to-size method, and 3) mother–child dyads using systematic sampling [24–26]. Mother-infant dyads fitting the age criteria were identified from a birth registry. Data were collected by face-to-face interview in a cross-sectional survey in 2011 in 11 provinces and another survey in 2012 in another province with the use of the same questionnaire and sampling strategy. We pooled data from the 12 provinces for this analysis. During recruitment, less than 5 % of the sample could not be reached because the mothers were not in town; they were replaced by an alternative in a pre-identified list. When reached, the response rate was 98 % and was similar for each ethnic group.

Using a structured questionnaire, we interviewed more than 11,000 mothers who belonged to 17 ethnicities. For this paper, we used data from 1875 mothers with children 0–23 months old who belonged to seven ethnicities that were collapsed to four ethnic groups: majority Kinh (*n =* 989; randomly selected from 9875 Kinh mothers, 10 % from each province), Thai-Muong (*n =* 309), Tay-Nung (*n =* 229), and E De-Mnong (*n =* 348). We did not include 163 mothers belonging to the other 10 ethnicities because they were too heterogeneous to be combined, and the sample size within each group was too small for a meaningful analysis.

### Outcome variables

IYCF practices were assessed using indicators recommended by the World Health Organization (WHO), based mainly on foods and drink consumed the previous day [13]. The four breastfeeding indicators were: 1) *early initiation of breastfeeding*, defined as the proportion of children born in the last 24 months who were put to the breast within 1 hour of birth; 2) *exclusive breastfeeding* (*EBF) under 6 months*, the proportion of infants 0–5 months of age who were fed exclusively with breast milk in the previous 24 h (no foods, no liquids with the exception of medications such as drops, syrups); 3) *predominant breastfeeding (PBF) under 6 months*, the proportion of infants 0–5 months of age who were fed predominantly with breast milk in the previous 24 h (similar to EBF but allowing plain water or non-energy liquids); and 4) *bottle feeding*, the proportion of children 0–23 months of age who were fed with a bottle with nipple [13]. In addition, we provided the prevalence of three other breastfeeding indicators: *continued breastfeeding at 1 year*, the proportion of children 12–15 months of age who were fed breast milk; *continued breastfeeding at 2 years,* the proportion of children 20–23 months of age who were fed breast milk [13]; and *prelacteal feeding practice*, the proportion of children 0–23 months of age who were fed with any foods or liquids other than breast milk to an infant during the first three days after birth.

Four WHO indicators for complementary feeding practices for children 6–23 months old were used: 1) *complementary feeding for 6–8 months old,* the proportion of infants aged 6–8 months of age received solid, semi-solid, or soft foods*;* 2) *minimum meal frequency,* the proportion of breastfed and non-breastfed children 6–23 months of age who received solid or semi-solid food (including milk feeds for non-breastfed children) the minimum number of times or more (eg, 2 times for breastfed infants 6–8 months, 3 times for breastfed children 9–23 months, and 4 times for non-breastfed children 6–23 months); 3) *minimum dietary diversity,* the proportion of children 6–23 months of age who received foods from 4 or more out of the 7 specified food groups; and 4) *minimum acceptable diet,* the proportion of children 6–23 months of age who received both minimum meal frequency and minimum dietary diversity, apart from breast milk [13].

### Main exposure variable

Self-identified ethnicity was defined based on direct interview with mothers. As mentioned earlier, we interviewed mothers belonging to 17 out of 54 ethnic groups in Vietnam and included seven ethnic groups (Kinh, Thai, Muong, Tay, Nung, E De, and Mnong) in the analysis. We then collapsed the seven ethnic groups into four (ie, Kinh, Thai-Muong, Tay-Nung, and E De-Mnong) based on the similarity of the ethnicities in geographic residences, economic characteristics, community organizations, marriage and family, and culture [14]. The ethnic majority Kinh served as the reference group.

### Covariates

Maternal age (18–24 years vs. ≥ 25 years), education (with ≤ 9 years – no high school vs. > 9 – some high school), and occupation were assessed. Family food-security status was estimated using the Household Food Insecurity Access Scale [27], and classified into: severe, moderate, and mild food insecurity, and food secure (reference group). Child age and gender were obtained from the face-to-face interview. We collected information about the place and mode of delivery and professional breastfeeding advice and support during pregnancy and during 3 days after birth.

### Statistical analysis

Analysis was performed using survey commands in Stata 13.1 (Stata Inc., TX, USA) to account for the sampling design with province, district (ie, stratum), and village (ie, primary sampling unit). Bivariate analyses were applied to assess the differences in maternal and household characteristics by ethnicity using two-sided chi-square test. The survey version of logistic regression that accounted for clustering was used to examine associations between ethnicity and specific breastfeeding and complementary feeding practices, adjusted for child age and gender; maternal age, education, and occupation; and household food insecurity. For early initiation of breastfeeding, we also controlled for cesarean delivery and professional breastfeeding advice and support during pregnancy and during 3 days after birth.

## Results

Ethnic minority women in this sample had younger age, lower education, greater participation in farming, and greater likelihood of experiencing food insecurity than the Kinh majority (Table 1). While deliveries occurred mostly in health facilities among the Kinh (98 %) and the Tay-Nung (97 %), facility deliveries were less prevalent among the E De-Mnong (~75 %) and Thai-Muong (~50 %). Cesarean section prevalence was 22 % among the Kinh, statistically higher than among the Tay-Nung (13 %), E De-Mnong (12 %), and Thai-Muong (6 %). Fewer ethnic minority mothers received professional breastfeeding advice during pregnancy and support during 3 days after birth than the majority Kinh.

**Table 1:** Child, maternal, household, and delivery characteristics by ethnicity^a^

	Kinh (*n =* 989)		Thai-Muong (*n =* 309)		Tay-Nung (*n =* 229)		E De-Mnong (*n =* 348)	
Maternal characteristics:								
Mother age (years):								
< 20 y	5.1	(3.9, 6.6)	17.2	(12.8, 22.5)*	9.6	(7.0, 13.1)*	14.1	(9.3, 20.7)*
20–24 y	32.0	(29.1, 34.9)	39.8	(31.4, 48.8)	46.3	(35.5, 57.4)*	34.5	(29.5, 39.8)
25–29 y	34.3	(31.1, 37.6)	28.8	(22.6, 35.9)	25.3	(20.5, 30.8)*	26.4	(22.4, 30.9)*
≥ 30 y	28.7	(26.0, 31.6)	14.2	(11.5, 17.5)*	18.8	(11.7, 28.8)	25.0	(19.6, 31.3)
Mother education level:								
≤ 5 y	10.2	(8.4, 12.4)	20.7	(8.9, 41.1)	27.9	(19.2, 38.8)*	39.9	(32.3, 48.1)*
6–9 y	49.7	(46.5, 53.0)	56.6	(49.2, 63.8)	41.5	(34.2, 49.2)	28.2	(24.2, 32.5)*
10–12 y	25.7	(23.0, 28.6)	14.2	(8.2, 23.5)	26.2	(19.0, 35.0)	29.9	(22.3, 38.7)
≥ 12 y	14.4	(12.4, 16.6)	8.4	(4.0, 17.0)	4.4	(2.3, 8.2)*	2.0	(0.9, 4.3)*
Mother occupation:								
Farmer, Fisherman	34.0	(30.5,37.6)	86.4	(81.5, 90.2)*	88.6	(82.7, 92.7)*	88.8	(85.5, 91.4)*
Salaried employee	21.1	(18.4,24.2)	7.4	(4.4, 12.2)*	3.5	(1.7, 7.2)*	2.9	(1.6, 5.1)
Small trader, self-employed	20.6	(18.2,23.3)	2.6	(1.4, 4.7)*	4.4	(2.5, 7.6)*	0.6	(0.1, 2.2)*
Household work/housewife	24.3	(21.4,27.4)	3.6	(2.7, 4.7)	3.5	(2.1, 5.7)*	7.8	(5.5, 10.9)*
Household food insecurity status:								
No (Food secured)	59.3	(55.6, 62.8)	26.2	(24.2, 28.3)*	27.5	(22.3, 33.4)*	11.5	(8.2, 15.8)*
Mid	14.6	(12.5, 16.9)	15.5	(11.0, 21.5)	22.3	(18.1, 27.0)	10.3	(7.6, 14.0)*
Moderate	18.8	(16.3, 21.6)	35.0	(28.2, 42.4)	44.5	(40.7, 48.5)*	50.9	(45.1, 56.6)*
Severe	7.4	(5.7, 9.5)	23.3	(13.9, 36.3)	5.7	(3.6, 8.9)*	27.3	(22.5, 32.7)
Delivery characteristics:								
Delivery venue:								
In hospitals	81.1	(77.5, 84.2)	23.9	(14.1, 37.6)*	73.4	(67.6, 78.5)	68.4	(56.4, 78.3)
In commune health centers	17.4	(14.5, 20.7)	27.2	(16.3, 41.8)	23.6	(17.5, 31.0)	7.5	(4.0, 13.7)*
At home	1.5	(0.6, 4.0)	48.9	(28.5, 69.6)*	3.1	(1.2, 7.4)	24.1	(15.7, 35.3)*
Cesarean delivery	22.4	(19.9, 25.2)	5.5	(3.2, 9.2)*	12.7	(9.6, 16.5)*	12.1	(8.1, 17.6)*
Professional breastfeeding advice or support:								
During pregnancy	47.9	(44.8, 51.1)	23.6	(17.8, 30.7)*	30.6	(23.4, 38.8)*	22.1	(17.3, 27.9)*
During 3 days after birth	32.6	(29.6, 35.7)	13.6	(11.9, 15.5)*	26.2	(21.7, 31.2)	18.4	(12.8, 25.7)*
Infant characteristics								
Being a boy	51.0	(47.9,54.0)	50.5	(46.9,54.0)	48.5	(43.8,53.2)	50.3	(44.5,56.1)
Mean age (mo)	8.5	(8.0,8.9)	10.4	(9.3,11.4)*	10.0	(9.4,10.7)*	7.7	(6.5,9.0)
Age groups:								
0–5 months	54.6	(51.2,58.0)	37.2	(34.6,39.9)*	39.7	(33.8,46.0)*	56.6	(45.1,67.4)
6–11 month	17.3	(15.1,19.7)	23.3	(16.0,32.6)	24.5	(21.3,28.0)*	19.0	(13.6,25.8)
12–17 months	14.1	(12.1,16.2)	23.0	(16.6,31.0)*	13.5	(9.3,19.3)	12.6	(8.7,18.0)
18–23 months	14.1	(12.2,16.2)	16.5	(12.5,21.4)	22.3	(19.2,25.6)	11.8	(8.5,16.0)

^a^Data from Alive & Thrive baseline surveys, 2011 and 2012 [24, 25]. Values are percentage (95 % CIs). Significantly different from Kinh (2-sided *χ* ^*2*^ test): *P < 0.05

### Ethnic variation in feeding practices in the first 3 days after birth

**Fig. 1:**
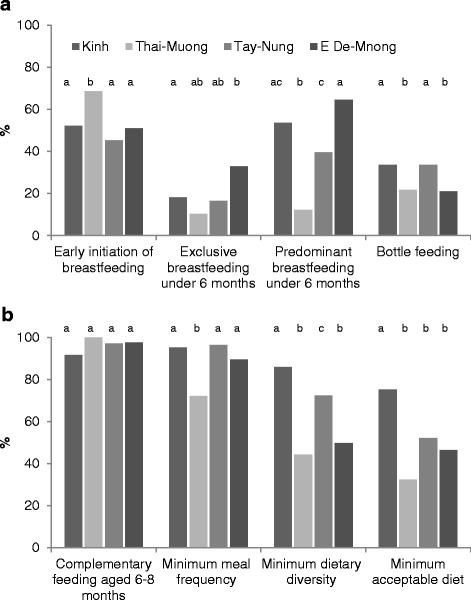
Select breastfeeding **a** and complementary feeding **b** practices by ethnicity. Data from Alive & Thrive baseline surveys, 2011 and 2012 [24, 25]. Values are percentages, *n =* 1875. ^a,b,c^ Different letters within an age group indicate statistically different; *P* < 0.05, two-sided *χ* ^2^ test. We used data from mothers with children 0–23 months old for early initiation of breastfeeding, prelacteal feeding, bottle feeding (*n =* 1875); 0–5 months old for exclusive and predominant breastfeeding (*n =* 943); children 6–8 months old (*n* = 189) for feeding of complementary feeding at 6–8 months; and children from 6 to 23 months old (*n =* 932) for other three complementary feeding practices

The prevalence of early initiation of breastfeeding among children born in the last 24 months was 69 % in Thai-Muong and about 50 % in the Kinh and the other two ethnicities (Fig. 1). The prevalence of prelacteal feeding was high (75–96 %) and differed by ethnicity. The type of prelacteal feeding also differed by ethnicity: Kinh newborns were mainly given infant formula (51 %) or plain water (49 %); Thai were mainly given herbal solution (53 %), chewed rice (44 %), and plain water (36 %); Tay-Nung were mainly given infant formula (59 %), plain water (43 %), and honey (37 %); and E De-Mnong were mainly given infant formula (51 %) and plain water (46 %).

In the logistic regression model, early initiation of breastfeeding was lower in the Tay-Nung (OR: 0.54; 95 % CI: 0.38, 0.77) than in the Kinh (Table 2). In addition, the prevalence was lower in mothers who had younger age, food insecurity, and cesarean delivery, but was higher in mothers who received professional breastfeeding advice and support during pregnancy and 3 days after birth, had a lower education, and were farmers (*P <* 0.05 for all).

**Table 2:** ORs (95 % CIs) for factors associated with selected breastfeeding practices by ethnicity in mothers with children 0–23 months old^a^

	Early initiation of breastfeeding (*n =* 1875)		Exclusive breastfeeding under 6 months (*n =* 943)		Predominant breastfeeding under 6 months(*n =* 943)		Bottle feeding(*n =* 1875)	
Mother ethnicity (ref. Kinh ethnicity):								
Thai-Muong	1.04	(0.72,1.51)	0.42	(0.25, 0.71)**	0.07	(0.01, 0.49)**	0.50	(0.37, 0.68)***
Tay-Nung	0.56	(0.39,0.80)**	0.67	(0.34, 1.32)	0.39	(0.19, 0.79)**	1.12	(0.75, 1.68)
E De-Mnong	0.75	(0.48,1.17)	1.99	(1.04, 3.82)*	1.35	(0.82, 2.24)	0.69	(0.50, 0.95)*
Mother age (18–24 y vs. ≥ 25 y)	0.66	(0.53,0.83)***	0.77	(0.50, 1.19)	0.75	(0.55, 1.02)	0.96	(0.72, 1.30)
Mother education (≤9 y vs. > 9 y)	1.30	(1.07,1.57)**	1.14	(0.75, 1.73)	0.86	(0.61, 1.23)	0.95	(0.64, 1.42)
Mother occupation (farmer vs. others)	1.40	(1.01,1.94)*	1.71	(1.18, 2.47)**	1.52	(1.03, 2.24)*	0.61	(0.39, 0.97)*
Food insecurity status (ref. food secured):								
Mild	0.66	(0.45,0.97)*	1.17	(0.60, 2.26)	1.05	(0.70, 1.59)	0.99	(0.64, 1.53)
Moderate	0.72	(0.57,0.91)**	0.81	(0.57, 1.14)	0.91	(0.65, 1.29)	0.96	(0.63, 1.46)
Severe	0.61	(0.45,0.81)***	0.77	(0.45, 1.31)	0.85	(0.50, 1.44)	1.10	(0.61, 1.98)
Child age (mo)			0.65	(0.59, 0.71)***	0.54	(0.48, 0.60)***	1.14	(1.02, 1.27)*
Being a boy	0.94	(0.75,1.18)	0.84	(0.55,1.27)	0.89	(0.68,1.17)	1.35	(0.88,2.06)
Delivery modes (ref. vaginal delivery out of hospital):								
Vaginal delivery in hospital	0.47	(0.31,0.71)***						
Cesarean delivery in hospital	0.03	(0.02,0.05)***						
Professional breastfeeding advice during pregnancy	1.27	(1.03,1.57)*						
Professional breastfeeding support during 3 days after birth	1.40	(1.10,1.78)**						

^a^Data from Alive & Thrive baseline surveys, 2011 and 2012 [24, 25]. Values are odds ratios (OR) and 95 % CIs. Significantly different from the null value (OR = 1; two-sided *t* tests): *P < 0.05, **P < 0.01, ***P < 0.001

### Ethnic variation in breastfeeding practices for children under 24 months old

The prevalence of EBF under 6 months was 18, 10, 17, and 33 % in the Kinh, Thai-Muong, Tay-Nung, and E De-Mnong, respectively. The prevalence of PBF under 6 months in Thai-Muong was 12 %, statistically lower than Kinh (54 %), Tay-Nung (40 %), and E De-Mnong (65 %). The difference in prevalence between EBF and PBF suggests that water was the main substance introduced (Fig. 1). The prevalence of bottle feeding was ~33 % in the Kinh and the Tay-Nung, statistically higher than that of the Thai-Muong and E De-Mnong (~20 %; Fig. 1). Bottles were used in most formula-fed children (75 % in Tay-Nung, ~80 % in Kinh and Thai-Muong, and 90 % in E De-Mnong). With children who were not fed infant formula, the bottle was used to feed other foods and drinks (28 % in Kinh, 20 % in Thai-Muong, 32 % in Tay-Nung, and 17 % in E De-Mnong). The prevalence of continued breastfeeding at one year was 79, 71, 71, and 90 % in the Kinh, Thai-Muong, Tay-Nung, and E De-Mnong, respectively. The prevalence of continued breastfeeding at 2 years dropped to less than 16 % for all ethnic groups except the E De-Mnong which was essentially unchanged from 1 year at 93 %.

Compared to the Kinh, the prevalence of EBF was lower in the Thai-Muong (OR: 0.42; 95 % CI: 0.25, 0.71) and higher in the E De-Mnong (OR: 1.99; 95 % CI: 1.04, 3.82). The prevalence of PBF was lower in the Thai-Muong (OR: 0.07; 95 % CI: 0.01, 0.49) and Tay-Nung (OR: 0.39; 95 % CI: 0.19, 0.79) than the Kinh. In addition, the prevalence of EBF and PBF was higher in mothers who were farmers than those involved in other occupations (Table 2). Bottle feeding was less common in the Thai-Muong (OR: 0.50; 95 % CI: 0.37, 0.68) and E De-Mnong (OR: 0.69; 95 % CI: 0.50, 0.95) compared with the Kinh (Table 2).

### Ethnic variation in complementary feeding practices for children 6–23 months old

Fewer ethnic minority children received minimum acceptable diets (33–52 %) than Kinh children (75 %, *P* < 0.05). Contributing to the low minimum acceptable diet was low minimum dietary diversity and meal frequency among the Thai-Muong, early discontinuation of breastfeeding among the Tay-Nung, and low minimum dietary diversity among the E De-Mnong (Fig. 1). Compared with Kinh mothers, ethnic minority mothers gave fewer legumes and nuts, dairy products (milk, yogurt, and cheese), flesh foods (meat, fish, poultry, and liver/organ meats), and vitamin-A rich fruits and vegetables (Additional file 1: Table S1).

Prevalence of minimum acceptable diet was lower in the Thai-Muong, Tay-Nung, and E De-Mnong than in the Kinh with OR of 0.23 (95 % CI: 0.11, 0.46), 0.52 (95 % CI: 0.39, 0.69), and 0.55 (95 % CI: 0.33, 0.89), respectively (Table 3). The lower prevalence was primarily due to lower minimum meal diversity in the Thai Muong (OR: 0.15; 95 % CI: 0.09, 0.27), Tay-Nung (OR: 0.59; 95 % CI: 0.39, 0.89), and E De-Mnong (OR: 0.26; 95 % CI: 0.16, 0.43), and lower minimum meal frequency in Thai-Muong (OR: 0.17; 95 % CI: 0.09, 0.30) (Table 3). Food insecurity was consistently associated with poorer complementary feeding practices (Table 3).

**Table 3:** ORs (95 % CIs) for factors associated with selected complementary feeding practices in mothers with children 6–23 months old^a^

	Complementary feeding aged 6–8 months (*n =* 189)		Minimum meal frequency (*n =* 932)		Minimum dietary diversity (*n =* 932)		Minimum acceptable diet (*n =* 932)	
Mother ethnicity (ref. Kinh ethnicity):								
Thai-Muong	-		0.17	(0.09, 0.30)***	0.15	(0.09, 0.27)***	0.23	(0.11, 0.46)***
Tay-Nung	2.65	(0.62, 11.43)	1.71	(0.70, 4.22)	0.59	(0.39, 0.89)*	0.52	(0.39, 0.69)***
E De-Mnong	2.21	(0.06, 76.27)	0.65	(0.39, 1.09)	0.26	(0.16, 0.43)***	0.55	(0.33, 0.89)*
Mother age (18–24 y vs. ≥ 25 y)	8.94	(1.35, 59.36)*	1.53	(1.04, 2.25)*	1.07	(0.69, 1.69)	1.12	(0.81, 1.56)
Mother education (≤9 y vs. > 9 y)	0.21	(0.04, 1.14)	0.79	(0.46, 1.38)	0.48	(0.33, 0.70)***	0.47	(0.31, 0.72)***
Mother occupation (farmer vs. others)	2.29	(0.28, 18.50)	0.72	(0.37, 1.40)	0.63	(0.40, 0.98)*	0.72	(0.52, 1.00)
Food insecurity status (ref. food secured):								
Mild	0.17	(0.02, 1.78)	0.58	(0.30, 1.11)	0.58	(0.39, 0.86)**	0.38	(0.29, 0.49)***
Moderate	0.84	(0.13, 5.54)	0.49	(0.31, 0.78)**	0.50	(0.32, 0.78)**	0.36	(0.25, 0.51)***
Severe	0.46	(0.01, 20.52)	0.43	(0.28, 0.66)***	0.31	(0.21, 0.44)***	0.26	(0.17, 0.40)***
Child age (mo)	6.85	(1.64, 28.65)**	1.01	(0.91, 1.12)	1.18	(1.09, 1.27)***	1.07	(1.03, 1.10)***
Being a boy	1.87	(0.50,6.98)	1.06	(0.77,1.46)	0.92	(0.66,1.29)	1.01	(0.70,1.46)

^a^Data from Alive & Thrive baseline surveys, 2011 and 2012 [24, 25]. Values are odds ratios (OR) and 95 % CIs. Significantly different from the null value (OR = 1; two-sided *t* tests): *P < 0.05, **P < 0.01, ***P < 0.001

## Discussion

In this study, IYCF practices were suboptimal and differed by ethnicity. Previous studies in Vietnam combined all ethnic minority groups and did not tease out differences among them [15–20]. For example, a previous assessment in Vietnam [17] showed that ethnic minority mothers had a higher prevalence of early initiation of breastfeeding than the Kinh majority (55 % vs. 37 %). Our study indicates that this may be the case in only some ethnic groups (eg, Thai-Muong) and not others (eg, Tay-Nung and E De-Mnong). Similar to previous studies [26, 28–32], we found that professional breastfeeding advice and support during pregnancy and after birth were associated with higher early initiation of breastfeeding practice. In addition to building capacity for health workers and improving baby-friendly environments at health facilities, building capacity of village health workers and traditional birth attendants who can provide breastfeeding advice and support is needed [15, 23, 32] because a large portion of ethnic minority mothers did not give birth at health facilities.

Prelacteal feeding practices also differed by ethnicity. Infant formula was the main prelacteal food for the newborn, which was found in previous studies in Vietnam [29] and other low-income countries [33–36]. Feeding infant formula in the 3 days after birth was common not only among the Kinh but also among some ethnic minority groups (eg, the Tay-Nung and E De-Mnong) who had very low food security, suggesting that formula companies might have expanded their reach to low-income and disadvantaged families in rural and mountainous areas. Previous studies with Vietnamese mothers in the country [29, 37] or who had migrated to high-income countries [9, 12] reported a perception that mothers after delivery need to rest, and thus would prefer having the newborn fed infant formula if available. Herbal solutions and chewed rice were the main prelacteal foods among the Thai-Muong while honey was common among the Tay-Nung. Feeding chewed rice to the newborn among the Thai-Muong has been reported previously in Vietnam [32] and Laos [38] to keep the newborn full [28, 32]. For certain ethnic groups in low- to high-income countries, herbal solutions are fed to enhance digestion or reduce fussiness [38–42], and honey is fed to avoid thrush and provide energy [39, 42] regardless of serious health risks such as botulism [43] and lead poisoning [40]. It is important to improve knowledge and self-efficacy through appropriate prenatal counseling and support. The messages should be consistently provided from the central to village level to shape beliefs and social norms toward more optimal IYCF practices.

The prevalence of EBF and PBF differed by ethnicity. Water and non-nutrient fruit juices were the main barriers to EBF for most ethnicities while early introduction of chewed rice was the main barrier to EBF in the Thai-Muong. Our findings indicate the need of ethnic-specific messages to improve EBF practices in Vietnamese mothers. A longitudinal study in Vietnam in 2002 [44] showed that the most common drinks for infants at weeks 16 and 24 were water (57.1 % and 90.4 %), fruit juice (14.7 and 19.4 %), and rice solution (5.0 and 24.4 %). The prevalence of using solid food was 40.9 % at week 16 and 74.3 % at week 24 [44]. The practices were driven by perceived breastmilk insufficiency, breastfeeding misperceptions (eg, formula was necessary with breastmilk insufficiency, complementary foods were good for health), and early return to work [28, 44].

The prevalence of bottle feeding in 2011 in our sample was 33 % [45], lower than that reported (39 %) in MICS 2011 [17], which might be attributed to the difference in sampling strategies. Nonetheless, it provides additional evidence to illustrate the high prevalence of bottle feeding in Vietnam, which has increased from 21 % in the early 2000s [46] to 39 % in 2011 [17] and 44 % in 2014 [20]. The use of infant-feeding bottles and artificial teats is associated with discontinuation of breastfeeding, diarrhea, impaired growth, infant mortality, and higher risk of overweight and diabetes [47–49]. Bottle feeding and non-EBF practices are particularly hazardous in communities with low access to improved water and sanitation such as rural or mountainous regions, low-income settings, disaster areas, and war zones [47]. The findings suggest the need for a nationwide intervention to minimize the use of bottles to feed formula and other foods and drinks.

Children belonging to an ethnic minority group had lower dietary diversity compared to Kinh children. Compared to Kinh children, ethnic minority children consumed fewer legumes and nuts, dairy products, vitamin-A-rich fruits, and vegetables (in all three ethnic minority groups); less animal foods and other fruits and vegetables (in the Tay-Nung and E De-Mnong); and fewer eggs (in the E De-Mnong). Kinh families typically live on a plain with a high population density and available markets [14]. In contrast, ethnic minority families typically live in mountainous or highland areas with a low population density and depend on subsistent or local foods [14, 44, 50]. Ethnic minority mothers were more likely to live in food-insecure families than Kinh mothers; food insecurity was associated with lower quality and quantity of complementary feeding. Food-insecure families tend to prioritize staple foods for current and future consumption, instead of diversifying their diets with nutritious foods [44, 50]. Food insecurity, however, is not the only factor associated with complementary feeding practices. For example, food insecurity was more prevalent, but complementary feeding practices tended to be better, in the Tay-Nung and E De-Mnong than in the Thai-Muong. This finding supports the potential of maximizing dietary quality even in food-insecure situations [51].

We used self-identified ethnicity obtained from an interview, and were not able to examine mixed ethnicity or acculturation towards the Kinh culture. In general in Vietnam, ethnicity is confounded with poverty and location (eg, some ethnic minority groups live in highlands or other places where there is high poverty) [14, 52]. To control this confounding, we included several aspects of socioeconomic status as covariates, and used districts as strata in the analysis. We did not collect information about exposure to mass media relating to breastfeeding and complementary feeding; the number of times that a mother received antenatal care, postnatal care, or exposure to breastfeeding and complementary feeding support after 3 days after birth; and family economic status, which limited our ability to separate the social and biological aspects of ethnicity.

## Conclusions

Breastfeeding practices were suboptimal and differed by ethnicity, which suggests the need for strong and tailored interventions at multiple levels to address ethnic-specific challenges and norms. Complementary feeding practices were less optimal among ethnic minority groups compared to the Kinh, which suggest the need for broad intervention, including improved food availability, access, and security. Together, these efforts have substantial potential to improve IYCF practices and lessen health inequity among different ethnicities in Vietnam.

The findings from this study are directly applicable to some other countries because the studied ethnic groups also live in neighboring countries (eg, Thai and Muong in Laos, Thailand, Southern China, Northeastern India, and Malaysia) or have migrated to other countries (eg, E De, Mnong, Thai, and Mong in the US). Furthermore, this study demonstrates that examining ethnic-specific IYCF practices in a given country provides important insights about IYCF; parallel research carried out in some of the many countries that also have distinct ethnic groups will further enhance understanding of the cultural basis for IYCF practices and ultimately how to help improve them.

## Abbreviations

DHS, Demographic and Health Surveys; EBF, exclusive breastfeeding; ISMS, Institute of Social and Medical Studies; IYCF, infant and young child feeding; MICS, multiple indicator cluster survey; PBF, predominant breastfeeding; WHO, World Health Organization

## Additional file

Additional file 1: Table S1. Type of foods consumed by 6–23-month-old children. (PDF 128 kb)
