# Supplementary material for: Infant and young child feeding practices differ by ethnicity of Vietnamese mothers
Source: BMC Pregnancy Childbirth. 2016 Aug 8;16:214. doi: 10.1186/s12884-016-0995-8 (PMC4977888; doi:10.1186/s12884-016-0995-8)
Supplement: Additional file 1: Table S1. — Type of foods consumed by 6–23-month-old children. (PDF 128 kb) [file 12884_2016_995_MOESM1_ESM.pdf]

Supplemental table 1 Type of foods consumed by 6–23-month-old children (%, 95% CIs)[1]

	Kinh (*n* = 449)	Thai-Muong (*n* = 194)	Tay-Nung (*n* = 138)	E De-Mnong (*n* = 151)
Type of foods (%):				
Grains, roots, and tubers	97.3 (95.2, 98.5)	94.8 (89.8, 97.5)	97.8 (93.9, 99.3)	98.0 (94.5, 99.3)
Legumes and nuts	43.0 (38.7, 47.4)	12.9 (6.9, 22.8)*	29.0 (25.9, 32.3)*	25.8 (18.8, 34.3)*
Dairy products (milk, yogurt, cheese)	84.2 (80.9, 87.0)	19.1 (9.8, 33.9)*	67.4 (62.9, 71.6)*	53.6 (46.9, 60.2)*
Flesh foods (meat, fish, poultry, and liver/organ meats)	86.2 (82.3, 89.4)	70.1 (46.8, 86.2)	72.5 (63.5, 79.9)*	59.6 (51.5, 67.2)*
Eggs	40.3 (35.6, 45.3)	43.8 (27.3, 61.8)	36.2 (28.3, 45.1)	27.8 (24.2, 31.8)*
Vitamin-A rich fruits and vegetables	84.4 (80.7, 87.5)	14.4 (6.7, 28.3)*	71.7 (58.0, 82.4)*	51.0 (40.8, 61.1)*
Other fruits and vegetables	67.7 (63.5, 71.7)	56.7 (34.8, 76.3)	51.4 (43.6, 59.2)*	33.8 (24.3, 44.8)*
By number of food group (%)				
0	0.7 (0.2, 2.1)	4.1 (2.7, 6.2)	1.4 (0.3, 5.9)	0.7 (0.1, 4.6)
1	3.6 (2.1, 6.0)	17.5 (4.7, 47.6)	8.0 (2.7, 21.4)	15.9 (10.8, 22.7)
2	3.3 (2.0, 5.4)	11.9 (6.3, 21.3)	8.7 (5.6, 13.2)	15.9 (9.7, 24.9)
3	6.5 (4.5, 9.2)	22.2 (14.8, 31.8)	9.4 (5.2, 16.4)	17.9 (13.5, 23.2)
4	16.7 (12.9, 21.4)	26.8 (17.3, 39.0)	21.0 (16.0, 27.1)	17.2 (11.2, 25.6)
5	26.5 (21.9, 31.7)	10.8 (5.6, 19.9)	26.1 (20.5, 32.6)	19.2 (11.9, 29.5)
6	25.2 (21.4, 29.4)	4.1 (1.5, 10.6)	19.6 (13.7, 27.1)	9.3 (5.9, 14.2)
7	17.6 (14.5, 21.1)	2.6 (1.1, 6.1)	5.8 (3.8, 8.7)	4.0 (2.5, 6.3)

[1] Data from Alive & Thrive baseline surveys, 2011 and 2012 [24, 25]. Values are percentages (95% CIs). Significantly different from the Kinh (2-sided χ^2 test): * $P < 0.05$.
